# Nutrition Awareness and Oral Health among Dental Patients in Palestine: A Cross-Sectional Study

**DOI:** 10.1155/2020/3472753

**Published:** 2020-02-26

**Authors:** Manal M. H. Badrasawi, Nour H. Hijjeh, Rana S. Amer, Reema M. Allan, Mohammad Altamimi

**Affiliations:** ^1^Department of Nutrition and Food Technology, Faculty of Agriculture and Veterinary Medicine, An-Najah National University, Nablus 30500, State of Palestine; ^2^Healthy and Therapeutic Nutrition Program, Faculty of Applied Sciences, Palestine Polytechnic University, Hebron 30500, State of Palestine

## Abstract

Nutrition plays a key role in oral and dental health. Similarly, oral health affects nutrition status and diet intake. Consumption of much cariogenic nutrients such as sugar affects dental and gum health. The awareness of dietary practices that affect the oral health is an essential component in the dental care system. The knowledge of the dietary factors that affect the oral health is a major component in the treatment plan. In this vein, this study was conducted to determine the level of awareness of nutrition information affecting oral health among dental patients who visit the private and university dental clinics in West Bank, Palestine. A total of 169 patients were invited to join the study and signed the consent form. A pretested questionnaire was used to collect the required data which included patients' sociodemographics, medical history, oral care practices, dietary practices, and oral health-related nutritional awareness. Face and content validity were verified, followed by a pilot study to determine the questionnaire reliability alpha, and the data were collected from October to November 2018. The Construct Validation was done using the Rasch measurement model, and the descriptive statistical analysis was done to determine the level of awareness and the difference among the groups using SPSS version 21. The total mean score of the nutrition awareness was (9.3 ± 2.8), with higher level of nutrition awareness among females, and the highest score was 16 out of 17. The good oral health condition was reported among 44%, fair 32%, poor 16%, and bad 10% while excellent oral health was reported among only 5% of the participants. There was no significant relationship between level of nutritional awareness with economic status, level of education, or area of living. For oral health, females showed significantly better oral and gum health levels (*p* < 0.05). The overall level of nutrition knowledge among the participants is insufficient. These results point to the need for oral health and nutrition educational courses and programs to improve oral and nutritional awareness and knowledge among Palestinian people and dental patients in particular. Improving the dietary habits and oral practices with lifestyle changes should also be encouraged.

## 1. Introduction

Oral diseases are the most common noncommunicable diseases (NCDs) spread in developed and developing countries. They are among the major public health problems worldwide that have affected half of the world's population (i.e., about 3.58 billion people) [1]. Such diseases include dental caries, periodontal disease, tooth loss, oral mucosal lesions, and oropharyngeal cancers. In addition to, human immunodeficiency virus/acquired immunodeficiency syndrome (HIV/AIDS) related oral disease and orodental trauma [1]. Oral health is an essential part in general health. It affects and is affected by nutritional status and overall health status. Moreover, it has an impact on the quality of life and health outcomes of the patient [2]. Poor oral health is associated with many chronic diseases such as diabetes mellitus, cardiovascular diseases, respiratory infections, and gastrointestinal pathologies. Moreover, poor oral health has been associated with low birth weight and preterm delivery [2]. On the other hand, good nutrition enhances healthy teeth and gum development and reduces the risk of some oral diseases [3]. Both macronutrients and micronutrients have an impact on oral health, teeth development, enamel and dentin synthesis, dental mineralization, and tooth protection [3].

Dental caries is a disease that results from microbiome dysbiosis with the involvement of multiple cariogenic species, including *mutans* streptococci (MS), lactobacilli, *Scardovia wiggsiae*, and several *Actinomyces* species that have the cariogenic traits of acid production and acid tolerance [4]. The relationship between nutrition and dental caries is a well-defined relationship; the anaerobic metabolism of dietary sugar by certain bacteria in the cavity leads to demineralization of enamel and dentin of the tooth by organic acid metabolites [5]. Depending on this mechanism, food items are categorized according to their effect on teeth into cariogenic, cariostatic, and anticariogenic food [3]. The carcinogenicity of food depends on bioavailability, digestibility, and texture of the consumed food [6]. Meaning that liquid food is less cariogenic than solid, while chewy food is less cariogenic than sticky ones [3, 5, 6]. Type of sugar also plays a specific role; that is, lactose has less effect than other sugars; and xylitol (alcohol sugar) reduces the risk of caries through its inhibitory effect on bacterial growth. Consumption of food and drinks that affect the oral cavity acidity (soft drinks, sweetened coffee and tea, candies) leads to teeth demineralization and causes tooth decay [7].

Previous findings have highlighted the preference of consuming fresh fruits to fruits juices because chewing stimulates more saliva production and promotes washing effect; and fruit juices may have extrinsic sugars and lower pH that contributes to erosive tooth wear. Concerning the meal patterns, it was reported that less frequent meals are recommended over more frequent meals due to their effect on dental caries; similarly, eating dessert after meals is preferred compared to eating them after a period of time [8].

Nutrition education on oral and dental health plays a vital role in preventing oral diseases and related problems. Hence, it should be included in management plans for any dental or oral problems. It maintains good oral health, enhances treatment outcomes, and prevents further dental problems. Higher nutritional knowledge is associated with better eating behaviors and with better nutritional status [9].

To the best of the researchers' knowledge, studies conducted to assess oral health nutrition related knowledge among dental patients or any age groups are very sparse. On the contrary, there are many studies conducted to assess the awareness, practices, and attitudes on oral health in general, without focusing on nutrition related knowledge among secondary school students [10, 11], primary schools students [12], housewives and mothers [13], and adults [14]. Some studies go further to assess the pediatricians' knowledge on dental caries prevention [15]. In Saudi Arabia, a knowledge, attitude, and practices study was conducted to explore the awareness level of oral health practices among adolescents age 10–18 years. It has found sufficient level of awareness without significant differences between males and females [16] while a study conducted on school teachers in India found that a fair level of oral health awareness was reported.

Having searched scientific databases including PubMed, Springer's, Science Direct, and Scopus, it was noticed that literature was lacking studies reporting knowledge or awareness of public or dental patients regarding the nutritional factors that affect oral health. A study was conducted in India to assess students' knowledge in nutrition and oral health relationship [8] and has found a low level of dental nutrition knowledge whose significance has differed according to the student's year of study. In Palestine, a study to address the relationship between oral health and nutritional knowledge among dental patients was recommended. Therefore, this study was designed to fill in such a gap by determining the levels of factors of oral health related nutritional knowledge.

## 2. Materials and Methods

### 2.1. Study Design

This study utilized the cross-sectional survey design. The main aim was to assess the level of nutrition and oral health awareness and to determine the relationship between nutritional status, oral practices, and oral health among adult patients.

The Al-Quds University Ethical committee approved the study protocol (reference number (55/REC/2018)). The researchers briefed the participants about the objectives of the study and gave them the right to participate in or withdraw from the study at any time. The researchers then obtained signed consent forms. The data collection has taken two months from October to November 2018. The data included participants' sociodemographics, medical history, oral health related practices, nutritional status assessments, oral health related nutrition knowledge, and oral health status.

### 2.2. Sample Size and Sampling Procedures

Sample size was calculated using G-power software for *t*-test with predetermined margin of error of 5% and confidence level of 95%. The required sample size was 120 participants. Thirty participants were added in case of the drop out given a total of 150 participants were recruited using the convenient sampling techniques. Eligible participants had to be adults aged 18–60 years and who had visited the dental clinic for treatment, scaling, or follow-up. The participants with congenital oral problems or under chemotherapy of radiotherapy were excluded from the final analysis. The selected participants were invited to fill in the questionnaire without referring to any information resources while answering the questions. No incentive was provided to the participants.

### 2.3. Research Instrument

#### 2.3.1. Nutritional Knowledge Questionnaire Development

The items of the questionnaire were developed based on the literature that reported the relationship between diet and oral health and oral diseases, in addition to the studies assessing the basic nutritional knowledge of an ordinary individual. The researcher with the help of three research assistants and an expert in the nutrition field prepared the first draft of the questionnaire which was composed of 20 items written in the participants' native language (i.e., the Arabic language). Nine experts in assessment, nutrition, and dentistry have verified the content and face validity of the questionnaire. A pilot study including 22 subjects from the private dental clinics in Hebron city was conducted to assess the internal reliability test of the questionnaire using SPSS™ version 21. Cronbach's alpha coefficient was 0.85, indicating the items have acceptable internal consistency. The final version of the questionnaire included 17 items. The items were presented as statements; and the subjects have to select if each statement is “correct,” “false,” or “I do not know.” In the analysis phase, the answers were coded as the following: (1 mark for the correct answer and 0 marks for the false or “I do not know” answers). The sum of the answers was calculated to get the total score out of seventeen for each subject. It is important to add that the psychometric properties of the 17-item questionnaire developed to assess the participants' nutritional knowledge were further examined using the Rasch Measurement model. According to Bond and Fox [17], Rasch measurement model is used for research instruments validation because it ensures that the research instruments have met the fundamental measurement requirements of research instrument, for both the whole instrument and the individual item as well as person [17]. The collected data were analyzed using the Winsteps software program, version 4.1.0 [18]. Overall, the Rasch analyses revealed that the questionnaire met the measurement requirements as reported in the results section below.

#### 2.3.2. Nutritional and Oral Health Status Assessment

Nutritional status was evaluated using anthropometric measurements, diet intake, and dietary practices. The anthropometric measurement included body weight and height, which were done following the standard protocol documented in Lee and Nieman [19]. The body mass index was calculated for each participant and the WHO cutoff points were used to categorize the nutritional status (obese, overweight, normal weight, and underweight) [19]. Food frequency questionnaire (FFQ) was used to assess the dietary intake. The questionnaire was validated in the Palestinian context (i.e., current study setting) by Hamdan et al. [20]. The questionnaire also included questions about nutritional practices to get detailed information about the eating patterns and food choices of the participants. The evaluation of overall oral health, oral hygiene, gum health, and presence of calcification was done by the dentists from the selected clinic. The rating score for oral hygiene, gum health, and overall oral health was done following the standard procedures from Carranza's Clinical Periodontology for oral health assessment using Periodontal examination chart which include Plaque index, Gingival index, mobility of the teeth, presence of calcification, and teeth furcation. The end score divided the oral health status into poor, fair, good, and excellent; for calcification, the assessment depends on the presence or absence of calcification [21].

### 2.4. Statistical Analysis

The Statistical Package for the Social Sciences SPSS™, version 21, was used to analyze the collected data [22]. The normality test was done for the nutritional knowledge scores using the Kolmogorov–Smirnov test. Descriptive analysis including the means and the standard deviations was used to analyze the data pertained to continuous dependent and independent variables. The categorical data were described by percentages. The independent samples *t*-test and ANOVA test were conducted to examine the differences in the total score of the nutritional knowledge due to selected independent variables at alpha <0.05. The Winsteps software program, version 4.1.0, was used to conduct the Rasch analysis to further examine the psychometric properties of the developed questionnaire on nutritional knowledge [23].

## 3. Results

### 3.1. Subjects' Characteristics

The total number of the participants invited and agreed to join the study was 169. Among them, 19 participants were excluded due to missing data. Only 150 participants were included in the final analysis.  Table 1 shows the demographic characteristics of the sample presented in frequencies and percentages.

The results revealed that smoking prevalence among males is higher compared to females (*p* < 0.05). Among the total sample, 13 patients reported presence of chronic diseases: 2 asthma, 5 diabetes, 4 hypertension, 1 thyroidism, and 1 heart disease. The cigarettes smokers' percentage is 52.1% of the total participants.

### 3.2. Participants' Oral Health Practices

Table 2 presents the oral care practices among the study sample. There was significant relationship between oral health practices and gender for the following items: “cleaning the teeth regularly,” “using teeth floss,” and “visiting the dentist for checking every 6 months.” The female participants recorded higher percentages in the aforementioned practices than the males. Females were found to use complementary or alternative methods such as using of salt, lemon, cloves, and cardamom in cleaning their teeth more as compared to males.

### 3.3. Dietary Habits

Dietary habits of the participants are presented in Table 3. There was significant relationship between dietary habits and gender only for “daily consumption of milk and its dairy products,” *p* < 0.05, where females tended to consume more milk daily and dairy products compared to males.

Table 4 presents the results of the participants' oral health status. Oral hygiene was significantly associated with gender, *p* < 0.05. The item “good” oral hygiene was higher in females than males. General oral health was also lower in males than females.

### 3.4. Nutrition Knowledge Score

The Rasch measurement model was used to examine the psychometric properties for the questionnaire developed to measure the participants' nutritional knowledge. Overall, the Rasch analyses showed that the research instrument has met the measurement requirements. The item and person reliability indices and unidimensionality through three indicators (item polarity, item fit, and PCA residuals) were examined as shown in Table 5. Table 5 shows that the reliability of item difficulty measures was very high (0.98) which indicates that replicability of the item difficulty ordering and the item separation index was 6.66, greater than > 2 [18]. Table 5 also shows that all the item polarities (i.e., point-measure correlation coefficients) are positive and ranged from 0.28 to 0.60, indicating that all the items are moving in the same direction to measure the intended construct [18]. The infit and outfit mean square statistics are also within the recommended range (0.5–1.5) [17], implying that the items are contributing meaningfully to measure the intended construct as expected by the model. However, the outfit square for item one is 1.52 due to misfitting persons. The Zstd values for both the infit and outfit statistics are within the range −2 to +2, except for infit statistics for item 1 (−3.2), which is still closer to the recommended range.

The principal component analysis of residuals is used to ensure if the data fit the model and items are measuring a single unidimensional construct.  Table 6 shows that unidimensionality is not violated. The variance explained by the measure is 45.3%, and the largest factor extracted from the residuals is equivalent to 2.0227 units which have the strength of about 2 items [18].

Overall, the participants showed inadequate knowledge about the specific dietary practices and oral health.  Table 7 demonstrates that the majority of participates answered the basic nutritional questions correctly; for example, 94.1% knew the answer for question 1, “food plays an important role in the teeth health,” and 93.3% gave the correct answer for question 10, “brushing or riddling the teeth after every meal reduces the decay.” On the other hand, the participants were not able to give the correct answers to other specific questions; for example, 84.4% wrongly answered question 17, “eating nuts with cariogenic foods protects the teeth,” and 76.3 % wrongly answered question 6; “increasing the number of the meals increases dental caries.”

The differences in the level of nutrition awareness and sociodemographic (age, gender, and marital status) were not significant. However, the monthly income shows significant relationship with awareness (*p* < 0.05). Patients with monthly income of more than 5000 have higher awareness scores. Awareness scores of females were also higher than those of males; however, no significance was noticed.

In terms of the relationship between nutrition awareness and oral health, there was no significant relationship between awareness level and oral health or oral hygiene.

Table 8 shows the relationship between the dietary intake with the oral and gum health. Participants with poor gum health have shown significant higher sugar intake while the relationship between oral health and sugar intake was found in patients with poor oral health; however, the relationship did not reach the significant level.

## 4. Discussion

This study aimed at assessing the level of nutritional awareness (related to oral health) and its relationship with oral and dental health among adult patients visiting dental clinics in Palestine. Overall, the results of the study revealed that the study sample lacked the essential nutritional knowledge indicated by their low scores on the given items regardless of their demographic characteristics. However, the level of income showed significant difference in the mean scores of nutritional knowledge, supported by the significant mean differences among the groups.

The overall mean score of the nutritional knowledge related to oral health was insufficient. The current study is unique because it has assessed nutritional knowledge in oral health, whereas many other studies have focused on knowledge, attitude, and practices related to oral health in general, mainly hygienic practices. A study conducted among nutrition and dietetic students in India highlighted low dental nutrition knowledge among nutrition/dietetics students [8]. This finding raised the issue that lacking nutrition knowledge related to oral health is a problem even among health professionals. By comparing the findings of this study with other studies focusing on oral health, almost similar trends were reported. For example, Szatco and his colleagues reported that Polish mothers lacked nutrition knowledge which affected adversely their kids' oral health [24]. Another study showed that Jordanian adults' poor level of knowledge related to periodontal diseases and the study recommended more educational programs to improve oral health awareness [25].

The study sample was comparably distributed according to gender: the study found no significant differences between males and females in the level of knowledge and awareness even though females usually recorded higher level of awareness and knowledge. Available literature reported a higher nutritional knowledge among females compared to males [26–29], and females showed more interest in nutrition and health, while no significant differences in nutrition knowledge and awareness levels between males and females were reported in Sarawak, Malaysia [10]. In terms of oral health, similar trend to the current study was reported; men showed less oral health knowledge compared to women [30]. In a study conducted in Palestine, significant gender differences were reported in knowledge attitude and practices of dental and oral health between males and females [31]. In Kuwait, poor oral health knowledge and practices among male students compared to female students were also reported [30].

### 4.1. Oral Health Status and Nutrition-Related Factors

The current study found that the relationship between nutrition knowledge and oral health was not significant. Participants with higher nutrition knowledge scores did not have better oral or gum health. However, “good oral health status” was significantly higher in females compared to males. In addition, “bad gum health” in males was significantly higher compared to females. Females reported better oral health compared to men, which may be due to the presence of other factors as smoking. Numerous studies have shown that tobacco use would lead to and increase incidences and severity of periodontal diseases and higher rates of tooth loss. The adverse effects of cigarette smoking and other forms of tobacco are numerous, and tobacco use has been associated with gingival, oral mucosa, and dental alterations [32]. Females gained higher scores on nutrition knowledge and got better practices related to oral health compared to males, which illustrate that females may be more interested in nutrition and oral health than men [33].

There were no significant differences between body mass index (BMI) and oral health, gum health, and oral hygiene. However, another study reported the association between body mass index (BMI) and oral health among adults; obesity and abdominal obesity were associated with increased prevalence of periodontal disease, while being underweight was associated with decreased prevalence of periodontal diseases. It concluded that obesity could be a potential risk factor for periodontal disease [34]. Chafee and Weston suggested a bidirectional relationship between obesity and periodontal diseases, as obesity, insulin resistance, and periodontal diseases all are accompanied with hyperinflammatory status, oxidative stress, and increased level of adipocytokines [35].

The frequencies and percentages of correct answers were variable among the items. General questions reported higher percentages of correct answers compared to oral nutrition specific items. Questions 1 and 10 asked about the role of food in oral health and brushing teeth preventing caries, respectively. Both items asked about basic general knowledge, which is well known to most of age groups. Similar findings were reported in different studies targeting different populations. Among school teachers in Saudi Arabia, satisfactory level of knowledge was found in general oral health questions such as oral hygiene practices [36]. Majority of school children was found to be familiar with the effects of oral hygiene on oral health [37].

However, high percentages of participants gave wrong answers to questions 2, 3, 4, 7, 12, and 13 asking about sugar and dental caries. Specifically, they were asked about the effect of sugars on dental caries, effect of other types of sweetener, frequency and the length of exposure between sugar and teeth, eating more frequent meals, and increasing the risk of caries by consuming sticky dessert which stays at the oral cavity for longer time. Such findings were surprisingly reported in Bapat et al., who found lack of knowledge of similar questions among nutritionist and dietetic students. [8].

Questions 11, 16, and 7 focused on protective dietary factors against dental caries (i.e., nutrients that play protective roles against dental caries if consumed with cariogenic food, including dairy products, nuts, and presence of protein or fat with sugars) [3]. The protective effect of dairy products got 84% of correct answers, while the other items reported much lower percentages of correct answers (31% and 14.8%). This variation may be due to the recommendations of drinking milk and dairy products for healthy teeth as a calcium source and not due to their awareness of effect of bioactive compounds and types of polypeptides found on milk and dairy products that have the carioprotective effect [38].

The questions on the role of salivary flow effect and the acidity of food received lower percentage of correct answers 26.7% and 40.7% though the questions are dealing with well-known documented effects. This may be due to lack of nutrition consultation in the dental clinic, or maybe dentists did not focus on dietary behavior that affects oral health and rather they only focused on oral hygienic practices.

The rest of questions 6, 8, 9, and 15 have asked about the relationship between food presence in oral cavity and tooth decay, including number of meals, food leftover inside the oral cavity, and dessert consumption immediately after meals [3]. The main concept for these 3 items was that the exposure between meals, mainly carbohydrate and sugars in the oral cavity, will increase the risk of decay. These results supported the main finding which was the lack in the specific oral health nutrition information among the study participants.

## 5. Conclusion

Overall, dental patients at dental clinics revealed insufficient nutrition knowledge related to oral health. The participants were unaware of the various aspects of oral nutrition, with the absence of the statistically significant differences between male and female patients. The results of the study recommend educational programs targeting the individuals visiting the dental clinics including all age groups to enhance their nutrition-related knowledge and nutrition practices, which may improve their oral health. The study suggests including oral health nutrition consultation regularly and continuously at dental clinics, schools, and any other possible places. Further research is needed utilizing different study designs (follow-up and longitudinal and intervention designs) to determine the effect of poor and good nutrition practices on oral health in short- and long-term practices. Further research is needed to determine effective intervention programs that can be implemented in the Palestinian dental clinics.

## Figures and Tables

**Table 1:** Participants sociodemographic characteristics presented in numbers and percentages.

Demographic characteristics		Number (*N*)	Percentage (%)
Gender	Male	44	32.8
	Female	90	67.2

Marital status	Single	60	44.8
	Married	70	52.2
	Divorced	3	2.2
	Widowed	1	0.7

Area of living	City	81	60.4
	Village	49	36.6
	Camp	4	3

Educational level	Secondary	51	38.1
	University	73	54.5
	Postgraduate studies	4	3

Monthly income	Less than 1500 NIS	26	19.4
	1500–3000 NIS	47	35.1
	3000–4500 NIS	29	21.6
	More than 5000 NIS	28	20.9

NIS: New Israeli Shekel.

**Table 2:** Oral health practices according to gender presented in *n* (%).

		Total		Male		Female		*p* value
		*n*	%	*n*	%	*n*	%	
Cleaning teeth regularly	Yes	86	64.7	19	14.3	67	50.2	0.00
	No	47	35.3	25	18.8	22	16.5	

Cleaning time	Morning	31	24.8	10	8	21	16.8	0.456
	Evening	17	13.6	8	6.4	9	7.2	
	Morning and evening	60	48.0	16	12.8	44	35.2	
	More than once a day	17	13.6	5	4.0	12	9.6	

Cleaning method	Circular	41	34.2	11	9.2	30	25.0	0.689
	Horizontal	34	28.3	9	7.5	25	20.8	
	Linear	28	23.3	9	7.5	19	15.8	
	Vibration	17	14.2	7	5.8	10	8.3	

Cleaning duration	Less than 2 min	61	49.2	22	17.7	39	31.5	0.159
	2-3 min	57	46.0	16	12.9	41	33.1	
	More than 5 min	6	4.8	0	0.0	6	4.8	

Visiting the dentist for checking every months	Yes	40	30.8	7	5.4	33	25.4	0.025
	No	90	69.2	34	26.2	56	43.1	

Visiting the dentist for cleaning every 6 months	Yes	27	20.9	4	3.1	23	17.8	0.060
	No	102	79.1	36	27.9	66	51.2	

Cleaning teeth after eating food and sweets	Always	26	19.4	8	6.0	18	13.4	0.052
	Often	22	16.4	3	2.2	19	14.2	
	Sometimes	43	32.1	13	9.7	30	22.4	
	Rarely	28	20.9	11	8.2	17	12.7	
	Never	15	11.2	9	6.7	6	4.5	

Using teeth floss	Always	11	8.3	1	0.8	10	7.6	0.056
	Often	8	6.1	0	0.0	8	6.1	
	Sometimes	20	15.2	6	4.5	14	10.6	
	Rarely	29	22	13	9.8	16	12.1	
	Never	64	48.5	24	18.2	40	30.3	

Using mouth wash regularly	Always	28	21.4	10	7.6	18	13.7	0.158
	Often	19	14.5	3	2.3	16	12.2	
	Sometimes	32	24.4	11	8.4	21	16.0	
	Rarely	24	18.3	5	3.8	19	14.5	
	Never	28	21.4	13	9.9	15	11.5	

Rubbing teeth with salt	Always	7	5.6	1	0.8	6	4.8	0.367
	Often	8	6.3	3	2.4	5	4.0	
	Sometimes	23	18.3	7	5.6	16	12.7	
	Rarely	16	12.7	3	2.4	13	10.3	
	Never	72	57.1	29	23.0	43	34.1	

Rubbing teeth with salt and lemon	Always	6	4.9	2	1.6	4	3.3	0.551
	Often	5	4.1	2	1.6	3	2.5	
	Sometimes	13	10.7	6	4.9	7	5.7	
	Rarely	13	10.7	2	1.6	11	9.0	
	Never	85	69.7	31	25.4	54	44.3	

Using coal	Always	4	3.4	1	0.8	3	2.5	0.834
	Often	0	0	0	0	0	0	
	Sometimes	4	3.4	2	1.7	2	1.7	
	Rarely	9	7.6	4	3.4	5	4.2	
	Never	102	85.7	35	29.4	67	56.3	

Using cloves	Always	10	8.1	2	1.6	8	6.5	0.129
	Often	6	4.8	1	0.8	5	4.0	
	Sometimes	16	12.9	3	2.4	13	10.5	
	Rarely	19	15.3	4	3.2	15	12.1	
	Never	73	58.9	31	25.0	42	33.9	

Using cardamom	Always	2	1.7	0	0.0	2	1.7	0.154
	Often	3	2.5	1	0.8	2	1.7	
	Sometimes	5	4.2	4	3.4	1	0.8	
	Rarely	14	11.9	3	2.5	11	9.3	
	Never	94	79.7	33	28.0	61	51.7	

**Table 3:** Dietary habits and practices according to gender presented in *n* (%).

		Total		Male		Female		*p* value
		*n*	%	*n*	%	*n*	%	
Are the meals consistent throughout the days	Always	15	11.2	5	3.7	10	7.5	0.727
	Often	51	38.1	17	12.7	34	25.4	
	Sometimes	34	25.4	10	7.5	24	17.9	
	Rarely	27	20.1	11	8.2	16	11.9	
	Never	7	5.2	1	0.7	6	4.5	

Do you eat fresh fruits daily (as whole or juiced)	Always	40	30.1	14	10.5	26	19.5	0.978
	Often	37	27.8	12	9.0	25	18.8	
	Sometimes	41	30.8	14	10.5	27	20.3	
	Rarely	12	9.0	3	2.3	9	6.8	
	Never	3	2.3	1	0.8	2	1.5	

Do you eat vegetables daily (as raw or cooked)	Always	47	35.6	16	12.1	31	23.5	0.983
	Often	32	24.2	11	8.3	21	15.9	
	Sometimes	34	25.8	10	7.6	24	18.2	
	Rarely	14	10.6	5	3.8	9	6.8	
	Never	5	3.8	2	1.5	3	2.3	

Do you eat brown bread	Always	16	12.0	4	3.0	12	9.0	0.258
	Often	16	12.0	3	2.3	13	9.8	
	Sometimes	25	18.8	12	9.0	13	9.8	
	Rarely	33	24.8	9	6.8	24	18.0	
	Never	43	32.3	16	12.0	27	20.3	

Do you eat processed meat	Always	6	4.5	4	3.0	2	1.5	0.216
	Often	10	7.5	2	1.5	8	6.0	
	Sometimes	34	25.4	14	10.4	20	14.9	
	Rarely	38	28.4	10	7.5	28	20.9	
	Never	46	34.3	14	10.4	32	23.9	

Do you eat fast food	Always	17	12.8	6	4.5	11	8.3	0.712
	Often	23	17.3	8	6.0	15	11.3	
	Sometimes	42	31.6	15	11.3	27	20.3	
	Rarely	32	24.1	7	5.3	25	18.8	
	Never	19	14.3	7	5.3	12	9.0	

Do you drink milk and its constitutes daily	Always	33	24.6	6	4.5	27	20.1	0.022
	Often	32	23.9	15	11.2	17	12.7	
	Sometimes	29	21.6	6	4.5	23	17.2	
	Rarely	19	14.2	10	7.5	9	6.7	
	Never	21	15.7	7	5.2	14	10.4	

Do you drink enough water (10 cups and more)	Always	38	28.8	12	9.1	26	19.7	0.776
	Often	35	26.5	13	9.8	22	16.7	
	Sometimes	41	31.1	14	10.6	27	20.5	
	Rarely	12	9.1	2	1.5	10	7.6	
	Never	6	4.5	2	1.5	4	3.0	

Do you watch your calorie intake within a meal	Always	9	6.8	2	1.5	7	5.3	0.386
	Often	14	10.6	3	2.3	11	8.3	
	Sometimes	21	15.9	6	4.5	15	11.4	
	Rarely	26	19.7	7	5.3	19	14.4	
	Never	62	47.0	26	19.7	36	27.3	

Do you eat nuts at least twice a week	Always	30	22.4	16	11.9	14	10.4	0.072
	Often	29	21.6	10	7.5	19	14.2	
	Sometimes	36	26.9	9	6.7	27	20.1	
	Rarely	28	20.9	7	5.2	21	15.7	
	Never	11	8.2	2	1.5	9	6.7	

Do you eat fish regularly twice a week	Always	15	11.2	8	6.0	7	5.2	0.233
	Often	15	11.2	7	5.2	8	6.0	
	Sometimes	30	22.4	9	6.7	12	15.7	
	Rarely	45	33.6	13	9.7	32	23.9	
	Never	29	21.6	7	5.2	22	15.4	

**Table 4:** Characteristics of oral health according to gender presented in *n* (%).

		Total		Male		Female		*p* value
		*n*	%	*n*	%	*n*	%	
Health of the gum	Bad	19	14.7	11	8.5	8	6.2	0.009
	Fair	36	27.9	14	10.9	22	17.1	
	Good	69	35.5	16	12.4	53	41.1	
	Excellent	5	3.9	0	0	5	3.9	

Calcification	Existed	69	53.5	29	22.5	40	31.0	0.008
	Not existed	60	46.5	12	9.3	48	37.2	

Oral hygiene	Poor	25	19.4	15	11.6	10	7.8	0.001
	Fair	37	28.7	14	10.9	23	17.8	
	Good	62	48.1	12	9.3	50	38.8	
	Excellent	5	3.9	0	0	5	3.9	

Oral health	Bad	10	7.8	7	5.4	3	2.3	0.004
	Poor	16	12.4	5	3.9	11	8.5	
	Fair	41	31.8	18	14	23	17.8	
	Good	57	44.2	10	7.8	47	36.4	
	Excellent	5	3.9	1	0.8	4	3.1	

**Table 5:** Items measures, fit statistics, and point-measure correlation coefficient.

No.	Item	Measure	S.E	Infit MNSQ	ZSTD	Outfit MNSQ	ZSTD	PT-measure CORR
1	Food plays an important role in the oral health	−3.36	0.46	1.20	0.6	1.52	0.9	0.29
2	Sugar is one of the food elements that causes dental caries	−2.25	0.31	1.08	0.4	0.90	−0.1	0.38
3	Chewing free-sugar gum reduces dental caries	0.88	0.20	0.99	−0.1	1.03	0.2	0.44
4	Using artificial sweeteners in candies and juices reduces the dental caries	1.84	0.23	1.01	0.1	0.85	−0.5	0.40
5	Drinking fruit juice cause dental caries more than eating the whole fruit	1.38	0.21	1.06	0.6	1.01	0.1	0.38
6	Increasing the number of the meals increases dental caries	1.9	0.23	1.08	0.7	0.99	0.1	0.35
7	The frequency of eating sugar is more cariogenic compared to the amount of the sugar	0.22	0.20	1.00	0.1	1	0.1	0.44
8	Food leftovers in the mouth are associated with dental caries	−2.45	0.33	1.02	0.2	0.9	−0.1	0.40
9	Eating sweets with meals is better than eating sweets between meals on dental caries	1.55	0.21	0.96	−0.4	0.95	−0.1	0.43
10	Brushing or flossing the teeth after every meal reduces the caries	−3.16	0.43	0.83	−0.4	0.64	−0.4	0.47
11	Drinking milk and dairy products protect the teeth from caries	−1.82	0.27	1.15	0.9	1.48	1.3	0.30
12	Eating sweets that melt in the mouth and swallowed faster are less harmful than the sweets that stay for a longer time in the mouth.	0.07	0.20	0.77	−3.2	−0.71	2.0	0.60
13	Sticky sweets increase the probability of teeth decay compared to melty sweets	−0.08	0.20	0.92	−1.1	−0.81	1.2	0.51
14	Foods that increase the saliva secretion increase the possibility for tooth decay	1.79	0.22	0.90	−0.8|	0.79	−0.8	0.46
15	Reduction of food eaten before bed helps protecting from dental caries	−0.45	0.20	0.91	−1.0	0.84	−0.9	0.50
16	The presence of proteins and fats with sugar-containing meals decrease the decay	1.38	0.21	1.18	1.8|	1.23	1.0	0.30
17	Eating nuts with tooth decay-causing foods protects the teeth from decay	2.55	0.27	1.03	0.3	1.35	1.1	0.28
Means		0.00	0.26	1.01	−0.1	1.00	0.−1	

*Reliability and separation*								
Item reliability		**0.98**						
Item separation		**6.66**						
Person reliability		0.71						
Person separation		1.57						

**Table 6:** Standardized residual variance in eigenvalue units (= item information units).

	Eigenvalue	Observed (%)	Expected (%)
Total raw variance in observations =	31.1017	100.0	100.0
Raw variance explained by measures =	14.1017	45.3	44.9
Raw variance explained by persons =	5.3887	17.3	17.2
Raw variance explained by items =	8.7129	28.0	27.7
Raw unexplained variance (total) =	17.0000	54.7 100.0	55.1
Unexplained variance in 1st contrast =	2.0227	6.5	11.9

**Table 7:** Frequencies and percentages of correct answers.

Knowledge items	T		F	
	*n*	(%)	*n*	(%)
1-Food plays an important role in the oral health.	127	94.1	7	5.2
2-Sugar is one of the most food element that cause dental caries.	119	88.1	15	11.1
3-Chewing free-sugar gum reduces dental caries.	54	40	80	59.3
4-Using artificial sweeteners in candies and juices reduces the dental caries.	32	23.7	102	75.6
5-Drinking fruit juice causes dental caries more than eating the whole fruit.	55	40.7	79	58.5
6-Increasing the number of the meals increases dental caries.	31	23	103	76.3
7-The frequency of eating sugar is more cariogenic compared to the amount of the sugar.	71	52.6	63	46.7
8-Food leftovers in the mouth are associated with dental caries.	121	89.6	13	9.6
9-Eating sweets with meals is better than eating sweets between meals on dental caries.	38	28.1	96	71.1
10-Brushing or flossing the teeth after every meal reduces the caries.	126	93.3	8	5.9
11-Drinking milk and dairy products protect the teeth from caries.	114	84.4	20	14.8
12-Eating sweets that melt in the mouth and swallowed faster is less harmful than the sweets that stay for a longer time in the mouth.	75	55.6	59	43.7
13-Sticky sweets increase the probability of teeth decay comparing to melty sweets.	79	58.5	55	40.7
14-Foods that increase the saliva secretion increase the possibility for tooth decay.	36	26.7	98	72.6
15-Reduction of food eaten before bed helps protecting from dental caries.	88	65.2	46	34.1
16-The presence of proteins and fats with sugar-containing meals decreases the decay.	42	31.1	92	68.1
17-Eating nuts with tooth decay-causing foods protects the teeth from decay.	20	14.8	114	84.4

**Table 8:** The relationship between oral health and oral hygiene and macronutrients, total calories, and sugar intake.

	Unit	Poor	Fair	Good	Excellent	*p* value
		Mean ± sd	Mean ± sd	Mean ± sd	Mean ± sd	
*Oral health*						
Total calories	Kcal/day	2750 ± 1722	1738 ± 1950	2650 ± 2149	1732 ± 1091	0.120
Fat	Gram/day	118 ± 75	80 ± 46	115 ± 97	106 ± 20	0.252
Carbohydrates	Gram/day	297 ± 204	167 ± 130	830 ± 3848	181 ± 98	0.742
Protein	Gram/day	37 ± 46	63 ± 55	132 ± 236	86 ± 83	0.189
Sugar intake	Gram/day	120 ± 87	58 ± 61	80 ± 76	87 ± 74	0.079

*Oral hygiene*						
Total calories	Kcal/day	2872 ± 1664	1576 ± 1004	2555 ± 2087	2253 ± 278	0.109
Fat	Gram/day	127 ± 70	71 ± 45	113 ± 92	103 ± 13	0.115
Carbohydrates	Gram/day	306 ± 202	156 ± 117	751 ± 3607	210 ± 61	0.821
Protein	Gram/day	37 ± 44	62 ± 51	122 ± 224	105 ± 55	0.295
Sugar intake	Gram/day	129 ± 84	83 ± 57	65 ± 76	60 ± 47	0.011

## Data Availability

The data are available upon request from the corresponding author.
